# GSK-3β, a pivotal kinase in Alzheimer disease

**DOI:** 10.3389/fnmol.2014.00046

**Published:** 2014-05-21

**Authors:** María Llorens-Marítin, Jerónimo Jurado, Félix Hernández, Jesús Ávila

**Affiliations:** ^1^Centro de Biología Molecular “Severo Ochoa”, Consejo Superior de Investigaciones Cientificas, Universidad Autónoma de MadridMadrid, Spain; ^2^Centro de Investigación Biomédica en Red sobre Enfermedades Neurodegenerativas, Instituto de Salud Carlos IIIMadrid, Spain; ^3^Biology Faculty, Autónoma UniversityMadrid, Spain

**Keywords:** GSK-3β, Alzheimer disease, kinase, neurodegeneration, tau proteins

## Abstract

Alzheimer disease (AD) is the most common form of age-related dementia. The etiology of AD is considered to be multifactorial as only a negligible percentage of cases have a familial or genetic origin. Glycogen synthase kinase-3 (GSK-3) is regarded as a critical molecular link between the two histopathological hallmarks of the disease, namely senile plaques and neurofibrillary tangles. In this review, we summarize current data regarding the involvement of this kinase in several aspects of AD development and progression, as well as key observations highlighting GSK-3 as one of the most relevant targets for AD treatment.

Alzheimer disease (AD) is a neurodegenerative disorder, first described by the German psychiatrist Alois Alzheimer in 1906. AD is the most common form of age-related dementia. The estimated annual incidence of this disease appears to increase exponentially with age, from approximately 53 new cases per 1,000 people between the ages of 65 and 74 to 231 new cases per 1,000 people over 85 (Hebert et al., 2001; Alzheimer’s Association, 2012). Although mostly unknown, the etiology of AD is considered to be multifactorial. Only a negligible percentage of cases have a familial origin, while most are linked to environmental, non-genetic risk factors of diverse nature (Blennow et al., 2006). AD is characterized by a progressive loss of episodic memory and by cognitive and behavioral impairments. The most relevant histopathological hallmarks of the disease are extracellular senile plaques composed by amyloid-β (Aβ) protein and neurofibrillary tangles (NFTs), the latter formed mainly by hyperphosphorylated tau protein.

The anatomical changes in AD are highly selective for certain brain areas, although alterations can be widespread at advanced stages of the disease. Nevertheless, as one of the most affected brain structures, the entorhinal cortex (EC) is considered an invariant focus of pathology in all cases (Van Hoesen et al., 1991). Anatomical studies have revealed that the EC gives rise to axons that bi-directionally interconnect the hippocampus and the rest of the cortex. Accordingly, it is widely accepted that the EC functions as a gateway to the hippocampus, a brain structure that plays a key role in memory acquisition and consolidation. The EC–hippocampus disconnection that occurs in AD is believed to play a prominent role in the aggravation of the memory impairments that characterize this neurodegenerative disease.

Glycogen synthase kinase-3 (GSK-3) is a highly conserved protein-serine/threonine kinase that was first isolated from skeletal muscle in 1980 as one of five enzymes capable of phosphorylating glycogen synthase (Embi et al., 1980). It was subsequently demonstrated that insulin triggers the inactivation of this kinase. In mammals, GSK-3 is encoded by two highly related genes encoding GSK-3α and GSK-3β, respectively. In the brain, GSK-3β regulates many crucial cellular processes, acting as a key switch that controls numerous signaling pathways (Doble and Woodgett, 2007; Forde and Dale, 2007). The dysregulation of this kinase occurs in the development of cancer, diabetes, AD, schizophrenia, and bipolar disorder, among others. Thus, given its relevance in pathophysiological processes, GSK-3 β is widely considered a therapeutic target of interest.

## GSK-3β AS A MOLECULAR LINK BETWEEN Aβ AND TAU

The Aβ peptide has been widely considered the cornerstone of AD pathogenesis, and its precursor protein APP is one of the most studied molecules in the field of AD research. The APP is a glycosylated surface membrane protein (Kang et al., 1987). Aβ is a cleavage product derived from the transmembrane domain of this large precursor protein. APP undergoes post-translational processing, involving cleavage by various secretases and proteases, via two major pathways. Firstly, in the non-amyloidogenic pathway, APP is sequentially cleaved by α- and γ-secretases, thus giving rise to easily degradable fragments. Three members of the α-disintegrin and metalloproteinase (ADAM) family (ADAM-10, ADAM-17, and ADAM-9) have been proposed to form the α-secretase complex (Buxbaum et al., 1998; Koike et al., 1999). GSK-3β may down-regulate the activity of this complex by inhibiting ADAM activity (Zhang et al., 2012). In addition to another three proteins (APH1, PEN2, and nicastrin), presenilin (PS) 1 and 2 function as the catalytic core of the γ-secretase complex. GSK-3β also regulates Aβ production by interfering with APP cleavage at the γ-secretase complex step, since both APP and PS1 are also substrates of this kinase (Cai et al., 2012). *In vitro* studies suggest that GSK-3β affects PS1 function, which is required for the generation of the toxic Aβ (Uemura et al., 2007).

In contrast, in the amyloidogenic pathway, APP is cleaved by β-secretase, generating a membrane-associated fragment (Zhang et al., 2012). Subsequently, γ-secretase releases Aβ, which tends to aggregate, giving rise to senile plaques and other insoluble oligomeric forms of the protein. The putative β-secretase, also known as β-site APP cleaving enzyme 1 (BACE1), is a type I transmembrane aspartyl protease whose active site is located on the luminal side of the membrane. The knock-down of *bace1* prevents Aβ generation and abolishes amyloid pathology in mice expressing the Swedish mutation of APP (Cai et al., 2001; Luo et al., 2001). The expression level and activity of BACE1 have been found to be elevated in AD patients (Holsinger et al., 2002). Accordingly, GSK-3β inhibition reduces BACE1-mediated cleavage of APP through a NF-κB signaling-mediated mechanism. This observation thus suggests that the inhibition of GSK-3β reduces Aβ pathology (Ly et al., 2013).

*In vitro* studies (Takashima et al., 1996b) and transgenic animal models of AD (Terwel et al., 2008) indicate that Aβ activates GSK-3β signaling (Takashima et al., 1996a, b) by preventing inhibitory phosphorylation of this enzyme in the case of *in vitro* studies and by an independent mechanism in the case of animal studies. A similar increase in GSK-3β activity has been observed in the brains of AD patients (Leroy et al., 2007). A feed-forward loop is established after GSK-3β pathological activation by Aβ, which subsequently contributes to abnormal APP processing and to synaptic failure (Deng et al., 2014). Consistent with this, GSK-3β inhibition has been shown to reduce Aβ production in AD murine models (Phiel et al., 2003; Rockenstein et al., 2007b) and to decrease Aβ-induced neurotoxicity in cultured neurons (Koh et al., 2008).

In post-mitotic neurons, the microtubule network is of particular significance in supporting axon function. Microtubule-associated proteins (MAPs) facilitate and regulate microtubule formation and stability. Tau is a MAP that is found mainly in the axonal compartment under physiological conditions. Tau associates with microtubules and stabilizes their polymerization. It has been suggested that the presence of tau is required for Aβ-induced toxicity (Rapoport et al., 2002; Santacruz et al., 2005; Roberson et al., 2007). NFTs comprise mainly hyperphosphorylated forms of tau protein. In contrast to normal tau, the hyperphosphorylated form of the protein acquires the shape of paired helical filaments (PHF-tau). Accumulating evidence indicates that the phosphorylated state of tau is closely associated with AD pathology (Augustinack et al., 2002). Accordingly, Aβ induces the formation of tau fibrils in culture (Ferrari et al., 2003). PHF-tau has been described to be an aggregated and insoluble deposit in the somatodendritic compartment (Gotz et al., 1995). In addition, this form of tau is often truncated at the C-terminal domain and is highly resistant to the action of phosphatases and proteases. While non-phosphorylated tau is a flexible protein, PHF-tau is an insoluble misfolded protein. During the course of NFT formation, tau progressively acquires a rigid conformation.

The distinct phosphorylation states of tau correspond to its physiological roles (Bretteville and Planel, 2008; Sergeant et al., 2008), and phosphorylation of some of its serine/threonine residues elicits a biological effect (Fuster-Matanzo et al., 2012). The three tau kinases, GSK-3β, CDK-5, and PKA, associate with both tau and microtubules. Although they show a wide spectrum of phosphorylation, the major phosphorylatable sites of tau for each kinase are limited in preference (Hashiguchi and Hashiguchi, 2013). Multisite phosphorylation occurs in PHF-tau and is explained by the catalytic activities of the different kinases, although the functional significance of this phenomenon is not completely understood. Indeed, a direct association of tau with GSK-3β takes place as a functional unit (Sun et al., 2002; Chun et al., 2004). Although GSK-3β phosphorylates at least 36 residues in tau (Hanger et al., 2007), the main phosphorylation sites identified for this kinase are Ser199, Thr231, Ser396, and Ser413 (Billingsley and Kincaid, 1997). A moderate phosphorylation of Ser46, Thr50, and Ser202/Thr205 has also been reported (Illenberger et al., 1998), and minor phosphorylation of other residues has been described (Hanger et al., 2007). A complete description of these phosphorylation sites is provided in an extensive review by Hashiguchi and Hashiguchi (2013).

In the pre-tangle stage of AD, scattered deposits of phospho-Thr231-tau are detected in the brains of patients (Luna-Munoz et al., 2007). Interestingly, similar to many other residues of tau, the phosphorylation of Thr231 demands the combined action of CDK-5 and GSK-3β (Li and Paudel, 2006; Li et al., 2006). GSK-3β requires a priming phosphorylation of this residue by other tau kinases. This phosphorylation reduces tau binding to microtubules (Sengupta et al., 1998). A similar mechanism has been described for Ser404 and other residues. Thus, the combined action of CDK-5 and GSK-3β seems to be required for the development of the epitope characteristics of PHF-tau (Plattner et al., 2006; Sengupta et al., 2006). Interestingly, the protein phosphatases PP-1 and PP-2 effectively dephosphorylate these sites, in such a way that the overall tau phosphorylation state is determined by the balance between kinase and phosphatase action. Subsequently, cleavage and conformational changes of tau occur after its phosphorylation. After neuronal cell death, intracellular NFTs are released into the extracellular space (Dickson et al., 1992). Interestingly, growing evidence indicates that hyperphosphorylated tau activates GSK-3β through an increase in oxidative stress, neuroinflammation, and apoptosis (Saeki et al., 2011). In addition, GSK-3β impairs lysosomal acidification, a process that entails an inadequate clearance of non-functional proteins (Avrahami et al., 2013).

In summary, increased GSK-3β activity has been used to model events occurring in AD, interventions that exacerbate cognitive impairments, and neuropathology in rodent models of AD (Gomez-Sintes et al., 2011). Conditional overexpression of GSK-3β in mouse hippocampal neurons results in impaired performance in the Morris water maze, hyperphosphorylation of tau, reactive astrogliosis and microgliosis, and neuronal death (Lucas et al., 2001; Hernandez et al., 2002). Restoring normal levels of GSK-3β activity reverses spatial memory deficits, reduces tau hyperphosphorylation, and decreases reactive gliosis and neuronal death (Engel et al., 2006). The deletion of tau in GSK-3β-overexpressing mice significantly ameliorates memory impairments, thus indicating that tau phosphorylation contributes to this cognitive impairment (Gomez de Barreda et al., 2010).

## PHYSIOLOGICAL AND PATHOLOGICAL REGULATION OF GSK-3β ACTIVITY

GSK-3β is constitutively active in most tissues and most commonly regulated by inhibitory phosphorylation on Ser9. GSK-3β can be phosphorylated on this serine by several kinases. This observation allows for an effective mechanism for several intracellular signaling pathways to control the activity of this kinase. However, the dysregulation of these signal transduction pathways results in failure to adequately repress GSK-3β, thus allowing GSK-3β to remain abnormally active. Such a status contributes to various pathologies, including neurodegenerative and mood disorders, diabetes, and cancer.

**FIGURE 1:**
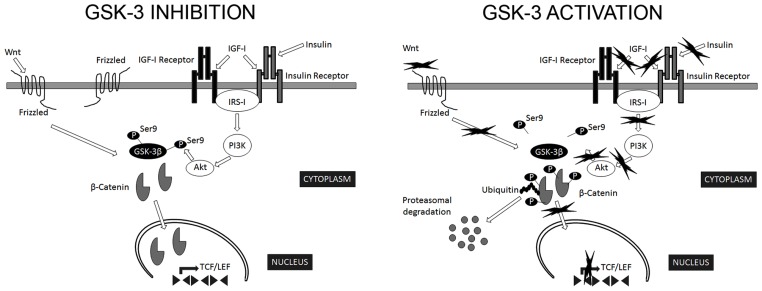
**Regulation of GSK-3β activity**. GSK-3β is constitutively active in most tissues and most commonly regulated by inhibitory phosphorylation on Ser9. The most relevant extracellular signaling pathways known to negatively regulate GSK-3β activity are those of insulin/insulin-like growth factor I and Wnt. In the canonical Wnt signaling pathway, Wnt stabilizes levels of β-catenin. Subsequently, stabilized β-catenin initiates the transcription of target genes. GSK-3β phosphorylates several components of this transduction pathway, β-catenin being the most widely characterized. Phosphorylated β-catenin is recognized by ubiquitin and targeted for proteasomal degradation (Wu and He, 2006). In addition, Akt phosphorylate Ser9 of GSK-3β in the context of insulin signaling pathway (Sutherland et al., 1993). Consequently, signals that modify GSK-3β activity are expected to alter β-catenin levels (Forde and Dale, 2007).

The most relevant extracellular signaling pathway known to regulate GSK-3β activity is that of insulin/insulin-like growth factor I (**Figure 1**). In addition, a number of kinases phosphorylate Ser9 of GSK-3β in the context of specific signaling pathways: PKB targets GSK-3β in response to insulin (Sutherland et al., 1993); PKA phosphorylates GSK-3β in Ser9 in response to cAMP (Cross et al., 1995); p90RSK/MAPKAP kinase-1 phosphorylates GSK-3β following activation by EGF or PDGF (Sutherland and Cohen, 1994; Fang et al., 2000); and p70S6K targets GSK-3β in response to stimulation by insulin and other growth factors (Godemann et al., 1999).

Interestingly, Aβ interferes not only with insulin but also with Wnt signaling pathways (Townsend et al., 2007; Magdesian et al., 2008). GSK-3β is a key transducer of the Wnt pathway (**Figure 1**), the components of which are involved in AD onset (Christian et al., 2002). It has been suggested that auto-inhibitory phosphorylation on Ser9 participates in the regulation of GSK-3β activity in response to Wnt (Saito et al., 1994; Fukumoto et al., 2001). In the canonical Wnt signaling pathway, Wnt stabilizes levels of β-catenin. Subsequently, stabilized β-catenin initiates the transcription of target genes. GSK-3β phosphorylates several components of this transduction pathway, β-catenin being the most widely characterized. Phosphorylated β-catenin is recognized by ubiquitin and targeted for proteasomal degradation (Wu and He, 2006). Consequently, signals that modify GSK-3β activity are expected to alter β-catenin levels (Forde and Dale, 2007). In addition, other components of Wnt signaling pathway, such as DKK1, negatively regulate these pathways, thus activating GSK-3β and contributing to the pathological events triggered by Aβ (Alvarez et al., 2004).

It has been proposed that GSK-3β activity also depends on the phosphorylation of Tyr216 (Kannan and Neuwald, 2004). The underlying mechanisms responsible for regulating tyrosine phosphorylation of GSK-3β remain controversial. In addition, it is still unclear whether GSK-3β autophosphorylation is an intramolecular or intermolecular event.

An interesting alternative regulatory mechanism of GSK-3β activity involves the action of the calcium-dependent protease calpain. GSK-3β is a calpain substrate (Goni-Oliver et al., 2007) and its cleavage by calpain produces the release of the inhibitory domain containing Ser9. Only in the GSK-3α isoform (but not in GSK-3β) is this region surrounded by glycine stretches, a feature that has been proposed to differentially regulate inhibitory phosphorylation and cleavage by calpain in both isoforms (Goni-Oliver et al., 2009).

## NEURAL CONSEQUENCES OF THE DYSREGULATION OF GSK-3β ACTIVITY

### CHOLINERGIC SYSTEM ALTERATION

A marked loss of cholinergic neurons in certain cortical areas is a well-known feature of AD brain (Whitehouse et al., 1981; Plotkin and Jarvik, 1986). It has been proposed that GSK-3β plays a key role in choline metabolism, which involves the regulation of choline acetyltransferase (ChAT) and acetylcholinesterase (Yates et al., 1983; Samadi et al., 2011). In fact, the loss of cholinergic neurons in the basal forebrain and hippocampus correlates with a transient decrease in Ser9 phosphorylation of GSK-3β and a concomitant increase in tau phosphorylation (Hoshi et al., 1996; Wang et al., 2009). In addition, cholinergic stimulation in the hippocampus, striatum, and cortex causes a rapid increase in Ser9 phosphorylation of GSK-3β (Wang et al., 2009).

### AXONAL TRANSPORT AND MICROTUBULE DYNAMICS IMPAIRMENT

Axonopathy and cytoskeletal disruption play a crucial role in AD (Kokubo et al., 2005; Robert and Mathuranath, 2007). GSK-3β has the capacity to phosphorylate several MAPs, thus regulating axonal stability through direct interaction with microtubules. GSK-3β-phosphorylated forms of tau and MAP-2 exhibit decreased affinity toward microtubules and are less stable (Lovestone et al., 1996; Sanchez et al., 2000; Zumbrunn et al., 2001). This microtubule destabilization is detrimental for the maintenance of axonal structure and appropriate synapse function (Sergeant et al., 2008). Importantly, Aβ plaques can lead to axonal dystrophy, causing profound impairment of axonal transport, great detriment to cognitive function, extensive synapse loss, and cell death (Rauk, 2008). Growing evidence indicates that axonal transport failure makes a significant contribution to AD pathology (Pope et al., 1993).

During neural development, GSK-3β is involved in axon formation and elongation (Bartzokis et al., 2003). In this regard, it impairs mitochondrial anterograde and retrograde axonal transport *in vitro*, a process that involves tau and MAP-1B, respectively (Jimenez-Mateos et al., 2006; Montenegro-Venegas et al., 2010; Llorens-Martin et al., 2011), and these alterations can have severe consequences on synapse function as a result of energy depletion. Accordingly, tau overexpression disrupts axonal transport, causing vesicular aggregation, a phenomenon reversed by GSK-3β inhibitors (Soutar et al., 2010).

In addition, PS1 regulates kinesin-related axonal transport by a mechanism involving GSK-3β activity (Ryan and Pimplikar, 2005) and the modulation of its role in controlling kinesin binding to microtubules at sites of vesicle release (Pigino et al., 2003).

### APOPTOSIS

Interestingly, GSK-3β promotes both pro-and anti-apoptotic effects. In this regard, it regulates the two major apoptotic pathways: intrinsic and extrinsic. GSK-3β triggers cell death through the activation of the mitochondrial intrinsic pro-apoptotic pathway while it inhibits the death receptor-mediated extrinsic apoptotic pathway (Beurel and Jope, 2006). After activation of the former, this kinase induces apoptosis in response to a wide range of detrimental stimuli, such as DNA damage (Watcharasit et al., 2003), hypoxia (Loberg et al., 2002), growth factor deprivation (Pap and Cooper, 1998; Johnson-Farley et al., 2006), and heat shock (Bijur et al., 2000). As a part of this pro-apoptotic cascade, GSK-3β phosphorylates and inhibits eIF2B (Welsh and Proud, 1993; Pap and Cooper, 2002). A murine model of neuronal GSK-3β overexpression developed by our group shows enhanced apoptosis in certain sensitive areas of the brain such as the hippocampal formation, which is crucial for memory and learning and strongly affected in AD (Fuster-Matanzo et al., 2011; Llorens-Martin et al., 2013). Although the exact mechanism by which GSK-3β overexpression induces apoptosis in these cells is unclear, it has been proposed that the combination of cell-autonomous effects and other effects indirectly mediated by inflammatory changes act in a coordinated manner to induce hippocampal neuron death (Llorens-Martin et al., 2013). However, given the regulation of the extrinsic apoptotic pathway by GSK-3β, it should be considered that this kinase modulates crucial steps in each of the two major pathways of apoptosis, but in opposing directions. Consequently, inhibitors of GSK-3β provide protection from intrinsic apoptotic signaling but potentiate that of extrinsic apoptosis (Gomez-Sintes et al., 2007). These observations should be taken into account when designing new therapeutic approaches and novel GSK-3β inhibitors.

### SYNAPTIC EFFECTS

Synaptic loss is currently the best neurobiological correlate of cognitive deficits in AD. In addition to the synapse loss caused by neuronal cell death, living neurons lose synapses in AD (Coleman and Yao, 2003). It has been proposed that the mechanism allowing information storage in the brain involves changes in synaptic connection weights, including long-term potentiation (LTP) and long-term depression (LTD). The finding that LTP inhibits GSK-3β activity and that this kinase is required for LTD suggests that LTP regulates LTD (Peineau et al., 2007). Although the exact mechanism underlying this regulation remains unclear, it has been demonstrated that constitutive GSK-3β activity enhances basal α-amino-3-hydroxy-5-methylisoxazole-4-propionic acid receptor (AMPAR) endocytosis (Wei et al., 2010). This phenomenon, leads to the dissociation of AMPAR-containing vesicles from kinesin (Du et al., 2010). In addition, tau and PS1 may be additional targets for GSK-3β regulation of synaptic plasticity, or, alternatively, different transcription factors or miRNAs may be involved in the protein synthesis-dependent phase of LTD (Manahan-Vaughan, 2010).

Of particular interest is whether the balance between LTP and LTD leads to functional impairments in memory storage similar to those described in AD (Bradley et al., 2012). LTD induces the removal of AMPARs from individual synapses (Luthi et al., 1999) in a process known as synapse silencing. These silent synapses are either reactivated through new AMPAR insertion (*unsilencing*; Isaac et al., 1995; Liao et al., 1995) or eliminated (Bastrikova et al., 2008; Egashira et al., 2010). Synapse elimination is particularly important during development and crucial for pruning unnecessary synaptic connections (Rabacchi et al., 1992; Bhatt et al., 2009). In fact, NMDAR-triggered apoptosis requires AMPAR endocytosis (Wang et al., 2004), a process known as *synaptosis*. In adulthood, *synaptosis* is down-regulated, and it is assumed that NMDAR-related LTD is used for adjusting synaptic weights rather than for eliminating synapses. Collingridge and colleagues suggested that neurodegeneration is often triggered by the reactivation of *synaptosis*, which leads to apoptosis of vulnerable neuronal populations (Bradley et al., 2012). The imbalance between these mechanisms may lead to the pathological elimination of synapses, which in turn leads to neuronal death. In this regard, we have demonstrated that neuronal GSK-3β overexpression causes a drastic decrease in postsynaptic density number and volume in hippocampal granule neurons (Llorens-Martin et al., 2013), a phenomenon that may be related to cognitive impairment and altered LTP generation previously observed in these mice (Hernandez et al., 2002; Hooper et al., 2007). In agreement, Aβ causes synaptic toxicity (Cullen et al., 1997; Witton et al., 2010). The use of GSK-3β inhibitors protects synapses from the deleterious effects of Aβ (Shipton et al., 2011), thus suggesting that GSK-3β activation is required for the pathological effect of Aβ on synaptic plasticity.

### INFLAMMATION

Among the functions regulated by GSK-3β, inflammation has recently emerged as one of the most relevant for neurodegenerative disorders (Sudduth et al., 2013). GSK-3β itself is an important positive regulator of the inflammatory process (Martin et al., 2005; Jope et al., 2007). Within the brain, microglial cells are considered to be equivalent to macrophages in the periphery and key guardian immune cells. Numerous stressors activate microglia, including neurodegenerative diseases, leading to a chronic inflammatory response and migration of responsive cells from the periphery. During long-term inflammatory responses, chronically activated (primed) glia appear to be detrimental to neuronal function and survival. Therefore, it is relevant that GSK-3β has been identified as a prominent regulator of inflammation. GSK-3β promotes the production of various pro-inflammatory cytokines, such as interleukin-6 (IL-6), IL-1β, and tumor necrosis factor (TNF; Martin et al., 2005). In addition, this kinase decreases the production of the anti-inflammatory cytokine IL-10. Remarkably, *in vivo* administration of GSK-3β inhibitors confers protection from endotoxin shock (Martin et al., 2005). Data from our group showed that GSK-3β overexpression in neurons leads to the appearance of a unique pattern of cytokines in the brain *in vivo* (Llorens-Martin et al., 2013). In addition, we have demonstrated that this pro-inflammatory environment is detrimental for immature neurons as it inhibits their appropriate maturation (Fuster-Matanzo et al., 2013) and leads them to acquire an aberrant morphology (named “V” shape) markedly similar to that found in AD patient granule neurons (Llorens-Martin et al., 2013).

### CELL CYCLE DYSREGULATION

The formation of dynamically re-arranged synaptic connections during continuous structural remodeling entails that neurons must permanently withdraw from the cell cycle (Arendt, 2003). As elegantly exposed in the “Dr. Jekyll and Mr. Hyde concept,” formulated by Arendt, after leaving the cell cycle, differentiated neurons modulate synaptic plasticity through molecular mechanisms primarily developed to control proliferation (Arendt, 2003, 2009). The up-regulation of a various molecular effectors involved in the activation and progression of the cell cycle occurs at early stages of neurodegeneration in AD (Arendt et al., 1996; Nagy et al., 1997a, b). Although the cause of this failure remains to be elucidated, recent evidence indicates that molecular mechanisms controlling synaptic plasticity and cell cycle are shared in the same cells, and, consequently, attempts to increase plasticity during initial stages of AD are sometimes disastrous for hippocampal function. At the molecular level, the Sonic hedgehog (Shh) and Wnt signaling pathways cooperate to orchestrate cellular proliferation, differentiation, and pattern formation during both development and adult neurogenesis. As previously discussed, GSK-3β plays a crucial role in modulating both pathways. Although the underlying mechanism regulating GSK-3β activity in response to Shh remains to be determined, Zhang et al. (2005) proposed the formation of a multi-protein complex similar to that required for efficient phosphorylation of β-catenin in the Wnt pathway. However, the physiological relevance of this interaction has yet to be revealed.

### ADULT HIPPOCAMPAL NEUROGENESIS

New neurons are continuously added to the hippocampal dentate gyrus (DG) throughout lifetime (Kempermann et al., 1998; Knoth et al., 2010). During differentiation stages, newborn neurons sequentially increase their dendritic tree complexity and send axons toward the CA3 region (Zhao et al., 2006). Growing evidence indicates that newborn neurons are crucial for hippocampal function and hippocampal-dependent memory (Bischofberger, 2007). One of the most important regulators of adult hippocampal neurogenesis (AHN) is GSK-3β. In this regard, it has been demonstrated that overexpression of this kinase impairs adult neurogenesis (Sirerol-Piquer et al., 2011; Fuster-Matanzo et al., 2013) and causes a depletion in the number of proliferative clusters within the hippocampal DG. In addition, we have recently reported that GSK-3β overexpression has dual effects on newborn neurons, blocking the differentiation of newborn neurons, thus supporting the notion that their maturation is impaired. We have observed that GSK-3β overexpression leads to alterations in the rate of death and survival of newborn neurons, as well as in the expression pattern of the immature neuron marker doublecortin (Fuster-Matanzo et al., 2013). In accordance, Spittaels et al. (2002) demonstrated that GSK-3β influences the post-natal maturation of neurons *in vivo* in a transgenic model overexpressing a constitutively active form of the enzyme. In addition, overexpression of this kinase causes morphological and connectivity alterations similar to those observed in the granule neurons of AD patients (Llorens-Martin et al., 2013). Given the relevance of newborn neurons in hippocampal-dependent learning, it is reasonable to assume that the alterations in AHN lead to cognitive impairments. In fact, murine model overexpressing GSK-3β in the hippocampus shows impaired hippocampal-dependent learning (Hernandez et al., 2002).

## AD THERAPIES INVOLVING GSK-3β INHIBITION

Growing evidence indicates that GSK-3β contributes to the pathology of several neurodegenerative diseases. Thus, there is increasing interest in applying GSK-3β inhibitors to treat these disorders. Lithium is a GSK-3 inhibitor that binds directly to GSK-3β (Klein and Melton, 1996) and increases the inhibitory phosphorylation in Ser9 of GSK-3β (Jope, 2003). Lithium is used as a mood stabilizer in patients suffering from mood disorders. Various effects of lithium are caused by GSK-3β inhibition (Jope, 2011), and lithium administration reduces the neuropathology and cognitive deficits in rats that have received intra-hippocampal injections of Aβ (De Ferrari et al., 2003), rats overexpressing GSK-3β (Liu et al., 2010), and several murine models overexpressing human APP (Rockenstein et al., 2007b; Ghosal et al., 2009; Toledo and Inestrosa, 2010). However, some studies report poor effects of lithium on behavior in other murine models of AD (Caccamo et al., 2007; Fiorentini et al., 2010; Sudduth et al., 2012). It is interesting to note that although certain cognitive tasks are improved by lithium treatment in healthy rodents, this metal does not significantly affect cognitive performance.

Recent years have witnessed the development of an increasing number of novel GSK-3β inhibitors, many of which are ATP-competitive. However, particularly promising are the non-ATP-competitive GSK-3β inhibitors, since they tend to be more selective and less toxic (King et al., 2014). The classical ATP-competitive GSK-3 inhibitors include Indirubin (Leclerc et al., 2001), Paullone compounds (Leost et al., 2000), SB415286 and SB216763 (Coghlan et al., 2000), and AR-A014418 (Bhat et al., 2003). Several well-known non-competitive ATP binding site inhibitors of GSK-3 are L803-mts (Plotkin et al., 2003; Kaidanovich-Beilin et al., 2004), TDZD-8 (Martinez et al., 2002), and VP0.7 (Palomo et al., 2011). The treatment of healthy rodents with GSK-3β inhibitors produces no remarkable effects on behavioral cognitive scores (Thotala et al., 2008). Conversely, genetic reduction of GSK-3β activity appears to be detrimental for hippocampal memory acquisition (Kimura et al., 2008). In contrast, GSK-3β overexpression (both the native and constitutively active forms of the enzyme) leads to cognitive impairment (Hernandez et al., 2002; Dewachter et al., 2009). In this regard, inhibitors of GSK-3β have been reported to rescue cognitive deficits in several murine models of AD. Treatment with NP12, AR-A014418, and Indirubin decreases memory deficits in the Morris water maze and reduces tau phosphorylation and amyloid deposition in various models of transgenic mice overexpressing human APP (Sereno et al., 2009; Ding et al., 2010; Ly et al., 2013). In addition, 5XFAD mice treated with L803-mts exhibit improved hippocampal-dependent learning capacity (Avrahami et al., 2013). Genetic approaches aimed to knock down either GSK-3α or GSK-3β have also been shown to improve cognitive impairments in several murine models of AD (Rockenstein et al., 2007a, b; Hurtado et al., 2012).

The promising ability of GSK-3 inhibitors to alleviate the AD-like phenotype of various murine models of AD has brought about several clinical studies in patients with this neurodegenerative disease, although contradictory data regarding the success of these treatments have been reported by different clinical trials (del Ser et al., 2013). It should be taken into account that GSK-3 is essential for cell life, and there is a concern that its inhibition could prevent cells from operating normally (Martinez et al., 2011).

Lithium has been shown to exert certain protection against the development of cognitive impairments in bipolar disorder patients (Nunes et al., 2007; Kessing et al., 2010). Importantly, patients in early-stage AD receiving lithium treatment showed improved cognitive function (Leyhe et al., 2009; Forlenza et al., 2011, 2012), although other studies showing no such enhancement have also been reported (Macdonald et al., 2008; Pomara, 2009).

## CONCLUSIONS AND FURTHER DIRECTIONS

GSK-3β is not a conventional kinase. It plays critical roles in neurodevelopment and in both physiological and pathological aging. In AD, a functional link between Aβ and tau unequivocally implicates the dysregulation of GSK-3β activity. In recent decades, Aβ was considered the cornerstone of AD etiology. However, the present consensus is that the disease has a multifactorial origin. Growing evidence supports inflammation as one of the most deleterious inputs to the aging brain. Given the relevance of GSK-3β in regulating crucial steps of the inflammatory cascade, efforts should be channeled into the development of novel and selective inhibitors that safely regulate the activity of this kinase, and, in parallel, block the inflammatory and self-propagating cascade that it triggers in previously damaged brain areas. Although the involvement of GSK-3β in multiple pathways controlling most of the crucial aspects of cell physiology complicates the design of specific inhibitors, it is of paramount importance to address the whole spectrum of GSK-3β actions on cell biology under both physiological and pathological conditions. A promising avenue are also regenerative strategies focused on the capacity of certain neural populations to be continuously generated and integrated into pre-existing neural circuits (adult neurogenesis). Given the pivotal role played by GSK-3β in the regulation of these processes, it is imperative to perform exhaustive research into the therapeutic potential of GSK-3β inhibitors. Such drugs would allow the normal development and functional integration of newborn neurons in the hippocampal formation previously damaged by the progression of the disease.

## Conflict of Interest Statement

The authors declare that the research was conducted in the absence of any commercial or financial relationships that could be construed as a potential conflict of interest.
